# A perspective on the use of the CatWalk device to assess dynamic spasticity in rodent models

**DOI:** 10.3389/fnins.2026.1857184

**Published:** 2026-08-05

**Authors:** Johannes C. Heinzel, Zachary Zamore, Sami H. Tuffaha

**Affiliations:** 1 Department of Plastic and Reconstructive Surgery, Johns Hopkins University School of Medicine, Baltimore, MD, United States; 2 Department of Hand-, Plastic, Reconstructive and Burn Surgery, BG Klinik Tuebingen, University of Tuebingen, Tuebingen, Germany

**Keywords:** animals, CatWalk, gait analysis, rodents, spasticity

## Abstract

**Introduction:**

Spasticity is a hallmark of upper motor neuron syndrome and arises from complex neurophysiological and biomechanical alterations, including disinhibition of the stretch reflex and secondary changes in muscle properties. While rodent models are widely used to study spasticity, objective assessment of dynamic spasticity during locomotion remains challenging. The CatWalk (CW) system is a well-established tool for automated gait analysis; however, its specific utility in quantifying spasticity-related gait abnormalities has not been systematically evaluated.

**Methods:**

A focused literature review was conducted to identify studies employing the CatWalk system in rodent models with features of spasticity. These parameters were then evaluated conceptually as candidate surrogate markers of dynamic spasticity and current evidence supporting their use.

**Results:**

Only a limited number of studies have explicitly used the CW system to assess spasticity-related gait alterations. A wide range of parameters was reported, including print-related, spatiotemporal and coordination-related parameters. While many parameters are sensitive to functional impairment, some might lack specificity for spasticity. In contrast, dynamic gait parameters such as stand phase duration, relative paw placement emerge as candidate surrogate markers but currently lack validation against established measures of spasticity.

**Discussion:**

The CW system offers substantial potential for assessing dynamic aspects of spastic gait in rodent models; however, careful selection and interpretation of parameters are critical. We propose potential candidates for future validation rather than established markers of spasticity. Future studies should focus on standardizing outcome measures and validating parameter sets that accurately reflect the underlying pathophysiology of spasticity, thereby improving translational relevance.

## 1 Introduction

Spasticity is a sequelae and major sign of upper motor neuron syndrome (UMNS) resulting from a complex interplay of neural and peripheral mechanisms that are not yet fully understood. Key contributors include impaired intraspinal processing of afferent input, loss of descending supraspinal inhibition—particularly of corticospinal origin—and increased excitability of motor neurons (Chen et al., 2025). The key feature of spasticity is the pathologically altered stretch reflex. Physiologically, the stretch (myotatic) reflex is a monosynaptic response to passive muscle stretch. Muscle spindles detect changes in muscle length and velocity, transmitting this information via fast-conducting group Ia afferents to the spinal cord. There, Ia afferents directly activate α-motor neurons of the agonist muscle while simultaneously engaging inhibitory interneurons to suppress antagonist activity, ensuring coordinated movement. γ-motor neurons further modulate spindle sensitivity and reflex gain. Following central nervous system injury, this tightly regulated system becomes disinhibited, resulting in a velocity-dependent exaggeration of the stretch reflex—the hallmark of spasticity. This imbalance is largely driven by reduced inhibitory input from the dorsal reticulospinal tract and relatively increased excitatory influences from the medial reticulospinal and vestibulospinal tracts (Ko et al., 2021). In addition to these neural alterations, secondary changes in muscle and tendon properties after upper motor neuron lesions further contribute to the clinical manifestation of spasticity and may limit functional recovery. Common mechanical alterations include reduced tendon compliance, increased passive muscle stiffness, and changes in extracellular matrix composition. Prolonged immobilization or maintenance of muscles in shortened positions leads to a reduction in the number of sarcomeres in series. While this adaptation accommodates the shortened muscle length, it also increases resistance to stretch and limits extensibility and range of motion (Reebye et al., 2024). Spasticity has a severe impact on affected patients, particularly regarding physical health and social capabilities (Barnes et al., 2017; Svensson et al., 2014; Vural et al., 2020). Besides being an area of clinical and experimental research in the vast field of neurology, the treatment of patients with spasticity is also a domain of plastic and reconstructive surgeons, reflected by a steadily growing body of research focusing on functional recovery (Aslami et al., 2026; Hurth et al., 2023; Orlando et al., 2023; Ran et al., 2024; Suresh et al., 2025). However, outcome measure in animal models of spasticity only seldomly include comprehensive gait analysis such as the CatWalk (CW) or comparable devices. The purpose of this Perspective is therefore not to establish CatWalk as a validated tool for measuring spasticity. Rather, we aim to highlight the current lack of evidence regarding which gait parameters, if any, specifically reflect dynamic spasticity and to discuss which parameters appear biologically most plausible for future validation studies.

## 2 Review of the literature

While there is a growing body of literature on the use of the CW device to assess gait in rodent models of peripheral (Heinzel et al., 2020a, c, 2021; Kappos et al., 2017) and central nervous injuries (Isvoranu et al., 2021; Timotius et al., 2023) as well as musculoskeletal disorders (Ritter et al., 2023), only few studies have specifically applied it to monitor signs of spasticity during walking (Table 1). Importantly, our search strategy intentionally focused on publications explicitly linking CatWalk with spasticity. The objective was not to summarize all CatWalk studies performed in upper motor neuron disease models but rather to evaluate the currently available evidence supporting CatWalk as a tool specifically for spasticity assessment. A PubMed query using the search terms “spasticity” and “catwalk” on April 1*^st^*, 2026 yielded a total of five studies underpinning the relatively small number of studies which have been conducted. Karadimas et al. (2013) used the CW in 2013 to investigate dynamic spasticity in rats with cervical spondylotic myelopathy and assessed hindlimb Base of Support (BoS), cadence, stride length, stand phase duration, swing speed, proportion of 4-limb support and relative paw placement. Following cervical spinal cord compression surgery, there was a gradual increase in BoS, which remained elevated over time. In parallel, stride length of all affected limbs decreased significantly, translating into an increased number of steps, i.e. cadence, required to traverse the walkway. In addition, interlimb coordination was markedly impaired, as evidenced by an increased relative paw placement, indicating a loss of the normal overlap pattern (Heinzel et al., 2020a). Dynamic gait parameters were also affected: stance phase duration and the proportion of four-limb support increased while swing speed was reduced. In summary and consistent with clinical observations, CW analysis demonstrated a progressive increase in base of support and a loss of coordinated, reciprocal hindlimb placement, thereby recapitulating key features of human spastic gait impairment, described as broad-based, ataxic, hesitant and jerky (Karadimas et al., 2013).

**TABLE 1:** Proposed candidate CatWalk parameters for dynamic spasticity.

Parameter	Biologic rationale	Current evidence	Major limitation
Print area	Functional impairment	Moderate (Hou et al., 2014; Pereira et al., 2024; Yang et al., 2021)	Unspecific
Walking speed	Overall locomotion	Limited (Hou et al., 2014; Van Gorp et al., 2013)	Strong speed dependence
Cadence	Overall locomotion/adaptation	Very limited (Karadimas et al., 2013)	Strong speed dependence
Stride length	Dynamic gait	Moderate (Karadimas et al., 2013; Van Gorp et al., 2013; Yang et al., 2021)	Unspecific
Stand duration/duty cycle	Stretch-related movement	Moderate (Hou et al., 2020; Karadimas et al., 2013; Pereira et al., 2024)	Not validated
Swing speed	Stretch-related movement	Moderate (Karadimas et al., 2013; Pereira et al., 2024; Yang et al., 2021)	Not validated
Stand index	Paw lift-off dynamics	Limited (Hou et al., 2014; Pereira et al., 2024)	Not validated
Base of Support (BoS)	Adaptation	Limited (Karadimas et al., 2013; Van Gorp et al., 2013)	Unspecific
Relative paw placement	Coordination	Limited (Karadimas et al., 2013; Van Gorp et al., 2013)	Unspecific
RI	Coordination	Limited (Hou et al., 2020; Van Gorp et al., 2013)	Unspecific
Phase dispersion	Coordination	Limited (Hou et al., 2014; Van Gorp et al., 2013)	Unspecific
Normal Step Sequence Patterns (NSSP)	Coordination/adaptation	None	Unspecific

Van Gorp et al. (2013) assessed runway crossing time, relative paw placement, hindlimb BoS, regularity index (RI), phase dispersions and stride length to measure spasticity in a rat model of acute lumbar spinal cord injury (SCI). While all parameters were affected following experimental surgery, only relative paw placements significantly improved in rats treated with human fetal spinal cord-derived neural stem cells (HSSC) compared to untreated SCI controls. No significant differences were observed in other CatWalk parameters or functional outcome measures between groups (Van Gorp et al., 2013).

In another rat model of cervical SCI, Hou et al. (2014) measured phase dispersions, walking speed, print area and stand index to quantify dynamic spasticity. Following cervical SCI, average walking speed and Stand Index were both significantly reduced compared to preinjury values in untreated animals and those which were treated with transcranial magnetic stimulation. Both parameters returned to levels not significantly different from baseline in animal which received treadmill training as well as a combination of the former and transcranial magnetic stimulation, indicating significant functional recovery with these interventions. Phase dispersions which reflect gait variability (Heinzel et al., 2020c; Timotius et al., 2023), were significantly increased after injury. Transcranial magnetic stimulation had no significant effect on phase dispersions; however, the parameter was not significantly different from preinjury levels in the group of animals which underwent treadmill training and combined treatment, respectively, suggesting normalization. In contrast, print area was significantly decreased after injury and did not show significant improvement with any treatment.

The same authors published a comprehensive analysis of gait behavior in the same model 6 years later (Hou et al., 2020), now analyzing the regularity index (RI), stand phase duration, initial dual stance, terminal dual stance and proportion of 4-limb support. The step sequence regularity index (SSRI) showed a significant deficit in untreated animals at post-injury time points, indicating disrupted inter-paw coordination, whereas the group treated with transcranial magnetic stimulation did not differ significantly from pre-injury levels. Similarly, the percentage of time standing on four paws was significantly increased in untreated animals postinjury while no significant difference was observed in the transcranial magnetic stimulation group compared to baseline. The initial dual stance duration, a marker of gait regularity and postural control, was also significantly increased in untreated animals at both time points, whereas animals treated with transcranial magnetic stimulation again showed no significant deviation from pre-injury values.

In 2021, Yang et al. (2021) measured print area, stride length and swing speed in a rat model of post-ischemic neuroinflammation due to middle cerebral artery occlusion. Postoperatively, CW analysis showed significant reductions in print area, stride length and swing speed compared to sham animals, in the authors’ opinion reflecting impaired gait due to ischemic limb dysfunction. Treatment with Gualou Guizhi Decoction (GLGZD) significantly improved all these parameters, indicating a measurable recovery of locomotor function (Yang et al., 2021).

Pereira et al. (2024) investigated the therapeutic impact of ultrasonic neuromodulation in a rat model spastic cerebral palsy using the CW method and analyzed swing speed, stand phase duration, swing time, stand index, print area and print width. Data was not reported groupwise and showed a significant level of heterogeneity between animals, therefore no definitive conclusion could be drawn as to which CW parameter(s) most accurately reflects the observed deficits due to spastic cerebral palsy, including dynamic spasticity during ambulation (Pereira et al., 2024).

## 3 Discussion

Spasticity is a cardinal symptom of multiple diseases of the central nervous system, which are commonly investigated with the CW device. However, only few studies have specifically investigated the CW’s ability to measure spasticity-specific changes of gait in small animals. This marks a relevant research gap in a time while both the incidence and relevance of spasticity and its respective treatment options grow rapidly (Aslami et al., 2026; Orlando et al., 2023; Ran et al., 2024; Suresh et al., 2025). A recent review has identified several clusters of gait parameters with high translational potential, i.e. spatiotemporal parameters, kinematics, kinetics and myoelectric signals (OuYang et al., 2025). While the CW device does not allow for assessment of comprehensive 3D data and ankle kinematics nor myoelectric signals, spatiotemporal parameters such as walking speed, stride length, swing time and cadence can be easily measured, all of which were identified as highly relevant to evaluate dynamic spasticity during walking (OuYang et al., 2025).

Importantly, none of the currently available CatWalk parameters has been systematically validated against accepted measures of spasticity such as H-reflex recordings, stretch-reflex electromyography, biomechanical resistance measurements (Bilchak et al., 2021) or quantitative clinical scoring systems adapted for rodents. Consequently, CatWalk cannot currently be regarded as a specific assessment tool for spasticity. Rather, the currently available evidence only allows discussion of biologically plausible candidate parameters whose relationship to spasticity remains to be established.

In our opinion, care must be taken to distinguish between the degree of sensitivity with which certain CW parameters may reflect spastic gait and the degree of specificity these parameters may change in animals with spasticity. This specificity could be determined by the extent to which the parameters reflect the impaired myotatic reflex. Circling back to the pathogenesis of spasticity, typical clinical manifestations include reduced tendon compliance, increased passive muscle stiffness and resistance to stretch as well as limited extendibility and range of motion (Kheder and Nair, 2012). In summary, spasticity causes a general impairment of the affected limb(s), which could, depending on the severity of the spasticity be reflected in a change of the so called general parameters of gait (Heinzel et al., 2020a), e.g., print area and print width. According to the literature, these parameters may indicate a general impairment of the spastic limb but are rather unspecific since, for example print area is altered by almost any kind of central or peripheral nervous injury or disease which results in decreased weight bearing on the affected extremity, respectively (Heinzel et al., 2020a; Ritter et al., 2023). Since the pathognomonic exaggeration of the myotatic reflex is directly related to the dynamic function of the affected limb, we therefore hypothesize that spasticity-related changes during ambulation may be best reflected by means of dynamic parameters of gait, i.e., swing speed, stand phase duration, relative paw placement, stride length and stand index. Especially Stand Index, i.e., the speed with which the paw loses contact with the represents a biologically plausible candidate that deserves prospective validation. The commonly assessed parameter Walking speed is highly variable (Batka et al., 2014; Deumens et al., 2014; Heinzel et al., 2020b) even in well-trained in animals and, since many CatWalk parameters are strongly influenced by locomotor speed, future validation studies should account for this important confounding factor. Walking speed, Although without a doubt affected by spasticity-related changes, might lack the required specificity and especially sensitivity to reflect spasticity. The same applies to the coordination-related parameters of RI and phase dispersions which are altered in a plethora of central nervous systems (CNS) diseases given that the central generator for locomotion is located within the CNS. Nevertheless, changes in RI or phase dispersions are likely rather unspecific for spasticity-related changes. Changes in BoS and the proportion of 4-limb support have been shown to compensate for unstable gait which can be of course attributed to spasticity but might also be a sequela of any disorder of the peripheral and central nervous system (Heinzel et al., 2020a). Finally, the analysis of the Normal Step Sequence Patterns (NSSP) might possibly offer valuable insights into how the animals adapt the use of the affected limb(s) following the onset of spasticity. It was shown that rats alter the use of their “preferred” NSSP following peripheral nerve injuries to offload the affected limb and these changes are at least partially reversed with the onset of functional recovery (Bozkurt et al., 2008; Heinzel et al., 2020c,^2021^; Ritter et al., 2025). Analyzing the distribution of NSSP before and after the onset of spasticity could therefore be used as complementary outcome measure to determine the effectiveness of treatments in rodents with spasticity.

From a mechanistic perspective, it is plausible that different CNS pathologies resulting in spasticity (Milligan et al., 2019) generate distinct CW signatures of spastic gait. Unilateral supraspinal lesions may be expected to predominantly affect parameters reflecting asymmetry, paw placement, and limb use imbalance, whereas SCI models may more strongly influence parameters related to coordination, timing, and abnormal stance-swing relationships. In disorders with fluctuating neurological involvement, such as multiple sclerosis, longitudinal variability may become an important feature. However, given the scarcity of studies specifically designed to correlate CatWalk outcomes with validated measures of spasticity, these considerations should currently be regarded as preliminary hypotheses rather than established model-specific patterns.

To enhance interpretability and translational value, CW-derived gait parameters should be systematically correlated with established measures of spasticity, including the Modified Ashworth Scale, quantitative muscle resistance measurements, electrophysiological readouts, and histological markers of reflex circuitry (Aslami et al., 2026).

Ultimately, the principal knowledge gap is not whether CatWalk detects locomotor impairment, which is well-established, but whether selected CatWalk parameters correlate with established neurophysiological measures of spasticity. Addressing this question should be considered a priority for future experimental studies.

In summary, CW-derived gait parameters differ markedly in their ability to reflect spasticity. Dynamic parameters appear biologically more plausible as candidate markers of dynamic spasticity but require systematic validation before conclusions regarding their specificity can be drawn. Focusing on these of gait may improve the dynamic metrics in addition to general parameters sensitivity and translational relevance of spasticity assessment in rodent models.

## Data Availability

The original contributions presented in this study are included in this article/supplementary material, further inquiries can be directed to the corresponding author.
